# Supplementary material for: Convergence and equilibrium in molecular dynamics simulations
Source: Commun Chem. 2024 Feb 7;7:26. doi: 10.1038/s42004-024-01114-5 (PMC10850365; doi:10.1038/s42004-024-01114-5)
Supplement: Supplementary file 2 — Supplementary Information [file 42004_2024_1114_MOESM2_ESM.pdf]

Supplementary Information
Convergence and Equilibrium in Molecular Dynamics Simulations

Franco Ormeño[1] and Ignacio J. General[2*]

[1]*Instituto de Investigaciones Biotecnológicas, Universidad Nacional de San Martín, San Martín, Buenos Aires, Argentina and*
[2]*Escuela de Ciencia y Tecnología, Universidad Nacional de San Martín, ICIFI and CONICET, San Martín, Buenos Aires, Argentina.*

I. SUPPLEMENTARY DISCUSSION

A. Dialanine

Considering the dynamical aspects of the very simple toy model of a protein, dialanine, panel A in Supplementary Figure 1 shows the COs of the first three normal modes. It can be seen that as more PCs are added, the COs increase, tending to a value of 1–as expected–but, more importantly, it's also observed that the longer the simulation, the more similar the curves are, with the longest four trajectories being virtually equivalent. This is showing that, in terms of global dynamical behavior, a dialanine trajectory longer that 100 ns can be considered converged.

Another metric that tests the dynamical behavior, again with respect to normal modes, is the one represented in panel B of Supplementary Figure 1; it shows the distribution of specific PCs for increasing time-lengths in the trajectory. We see a slightly off convergence at 100 ns, probably negligible for practical purposes, which turns almost perfect at 1 μs.

To test for structural convergence, conformations along the whole trajectory were grouped in a few clusters, according to their RMSD values between pairs of conformations, forming five relevant clusters. Panel C in Supplementary Figure 1 displays the corresponding cumulative fractional population for each cluster (left plot), and the cluster identity of each frame (right plot). This figure shows that there are three main clusters (0, 1 and 2), which are thoroughly explored, with many transitions between them, resulting in a clear convergence of their cumulative populations. The location of the clusters is shown with numbers and arrows in the free energy plot (bottom/right panel) of Figure 1. The three most populated ones, 0, 1 and 2, represent the deep wells in free energy (notice cluster 1 combines two wells, since they are closer than some limit distance used in the calculation), and clusters 3 and 4 represent the small ones located at the positive ϕ region. Supplementary Figure 1, panel C, also shows the previously mentioned fact of the different convergence rates in a given system: while clusters 0, 1 and 2 are heavily visited along the trajectory, with many transitions between them, the remaining two clusters, 3 and 4, only show a few events. This points to a highly converged sampling of the former group of clusters, from where statistically relevant conclusions can be extracted, while the latter group cannot be considered converged by any means.

And to end this assessment of dialanine's structural and dynamical convergence, Panel D in Supplementary Figure 1 displays the RMSF curves obtained from increasing lengths of the trajectory. Similarly to the graphs in Panel A, we observe that the RMSF of trajectories longer than 10 ns are almost perfectly converged.

B. Trp-Cage

Trp-cage is a 20 aminoacid miniprotein, originally designed by Neidigh et al. [1] which, due to its size and fast folding rate, is used as a test system for both experimental and computational studies of the folding/unfolding dynamics. Supplementary Figure 2 shows the results of our simulations. Panels A presents the cumulative overlap of ANM vs PC modes, with very good convergence in the first three modes starting at 10 ns except, perhaps, for mode 2 which needs some more time to become practically indistinguishable from the longer trajectories. Similarly, panel D shows almost perfect convergence for times above 1 μs, but it is already very good for 100 ns. Panel B compares the convergence of PC2's distribution for all the trajectory (which includes 4 μs of unfolded conformations) with that of the folded case, to stress the importance of removing the un-equilibrated initial part of the trajectory when analyzing convergence. This type of cumulative measurements, like PC distribution or ACFs, keeps track of those non-equilibrium origins, taking longer in order to express the convergence. Here, the full trajectory distribution does not seem to achieve

* igeneral@unsam.edu.ar

convergence, while the equilibrated one does achieve it by 1 μs. Finally, panel C also shows very good convergence. The initial conformational exploration is visible in the other 4 clusters, during the first 2.5 μs, but then it fully settles to cluster 0. In summary, Trp-cage, with the caveat of excluding the unfolded part of the simulation, shows an almost ideal convergence.

C. VHP

The next system is the 35 residue headpiece domain of the Villin-1 actin-binding protein (VHP). As Trp-cage, and due to its small size and fast folding rate, it is usually used as a test system in MD simulations. Based on a 22 μs trajectory, Supplementary Figure 3 shows very well converged properties of VHP. In particular, panel B contrasts the frequency distributions of PC2, considering all residues, and ignoring the terminal ones. As previously commented, terminal residues tend to be very flexible and, thus, tend to require longer times to reach convergence. But this does not affect the equilibrium properties of the central aminoacids. In the present case, it can be seen that the latter plot already displays excellent convergence at 1 μs. The same conclusion is extracted from the other panels.

D. GAAC

The trajectory of the GAAC (DNA duplex with sequence GCACGAACGAACGAACGC), was thoroughly analyzed by Galindo-Murillo *et al.* [2], with their findings pointing to an excellent convergence of the system, especially when ignoring the terminal base-pairs, which are much more flexible than the central core of the molecule, and present many chaotic fraying events. Supplementary Figure 4 shows the results from our present analysis, completely agreeing with that study, and showing full convergence for the whole system (including terminal pairs), with the exception of PCs histograms (panel B shows PCs 1 and 2 for the case for the 10 central pairs). It can be concluded that full convergence for the central part is already achieved by 1 μs.

E. Barnase

Supplementary Figure 5 shows the results for the 199 aminoacid long barnase protein. Panels A and D present results similar to the ones of dialanine, only that now the convergence starts later, on the order of the microsecond, and is highly converged at 10 μs. The cumulative population of clusters (Panel C) also shows a strong convergence of the whole trajectory. At first sight, the cluster distribution in Panel C appears to question this convergence, as all clusters–except for number 0–have a non-uniform distribution in time. As discussed in relation to dialanine, that is not something desirable if the goal is to calculate probabilities of the non-uniformly distributed states. But, in general, the aim of the majority of studies in MD is to characterize the most stable conformations, and calculate properties intrinsic to them (as opposed to transitions between them). And this can be confidently done as long as those clusters are visited a statistically significant amount of time. In view of the cumulative population plot, clusters 0 and 1 are, indeed, significantly visited, and we could safely calculate their properties.

Panel B displays the density of PC2 values for different lengths of the trajectory, with the left part representing all the residues in the system, while the right part shows only the contribution of central residues, excluding the termini, and only considering the backbone atoms. We see a clear difference between them, where the density for all residues does not appear to converge too well, but the other one does, for the longest times; in the latter, trajectories with 10 and 15 μs are virtually identical. This is, again, showing that the question of convergence should be considered in terms of specific behaviors, since different properties have different convergence times. In this case, the backbone reaches a stable distribution of values for PC2, while the side-chains would still need more time. A similar outcome is observed for the rest of the first five PCs of barnase.

F. Elastase

The last analyzed system is the 274 residue elastase protease. Overall convergence is mostly achieved, as observed in Supplementary Figure 6. Nevertheless, panel B shows PC1 having a slight non-convergence when going from 5 to 9 μs; during that time the peak around -30 tends to disappear, and the non-global maxima slightly displace to the right. The overall shape of the histogram and the location of the global maximum is still the same though. In terms of clusters, it can be concluded from panel C that cluster 0 is clearly the most populated one, and cluster 2 follows

far away, but still with a uniform population and many visits all along the trajectory, making for a good convergence of the intercluster transition rate.

G. Trajectories

Movies of each trajectory of the eight simulated systems can be found as Supplementary Movies 1 through 8. And pdb files with the first and last frames of each simulation are also available as Supplementary Data 1 through 16.

SUPPLEMENTARY REFERENCES

[1] J. W. Neidigh, R. M. Fesinmeyer, N. H. Andersen, Designing a 20-residue protein, Nature Structural Biology 9 (6) (2002) 425–430. doi:10.1038/nsb798.
URL https://doi.org/10.1038/nsb798

[2] R. Galindo-Murillo, D. R. Roe, T. E. Cheatham, Convergence and reproducibility in molecular dynamics simulations of the dna duplex d(gcacgaacgaacgaacgc), Biochimica et Biophysica Acta - General Subjects 1850 (2015) 1041–1058. doi:10.1016/j.bbagen.2014.09.007.

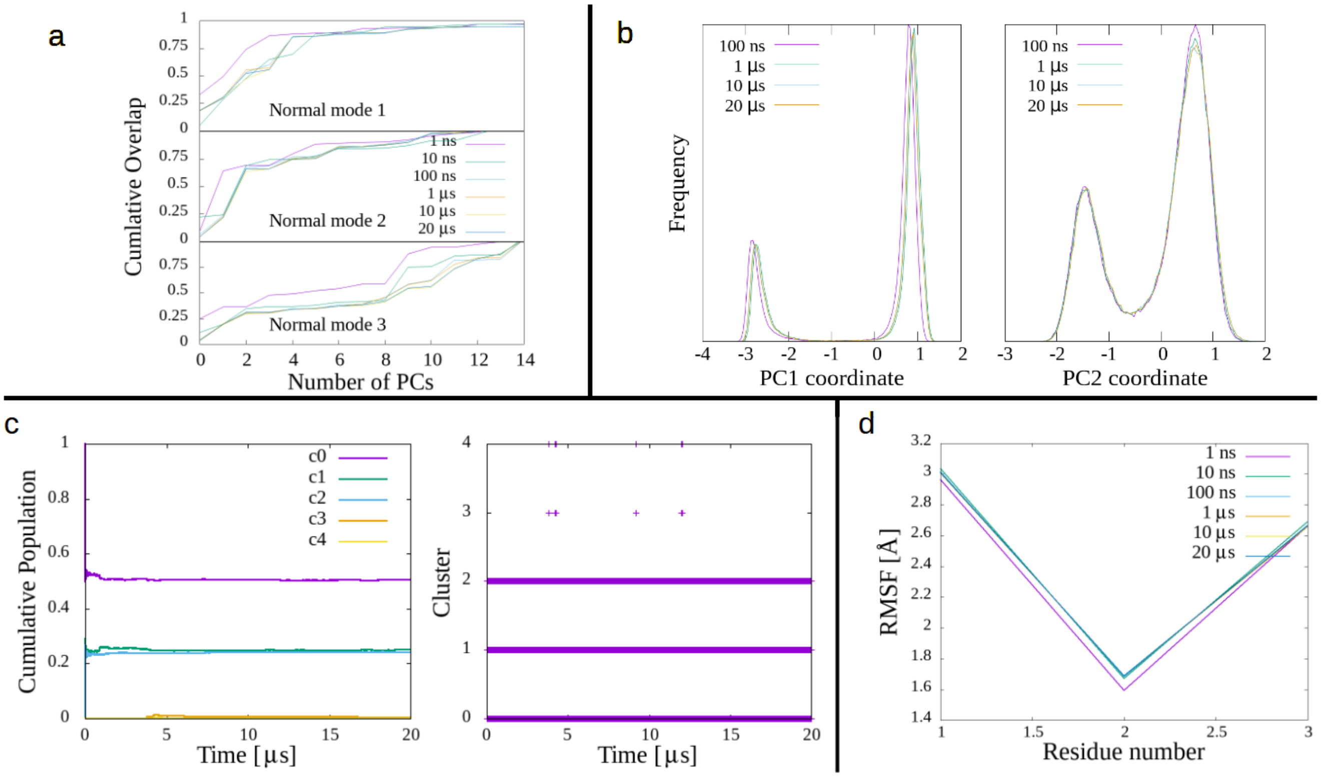

Supplementary Figure 1. Metrics of convergence in dialanine. A) Cumulative overlap of first 3 three ANM modes, in terms of MD PCs, for several simulation times. B) Frequency distribution of selected PCs for several simulation times. C) Cluster cumulative population (left) and time-distribution (right). D) Residue root-mean square fluctuations for several simulation times.

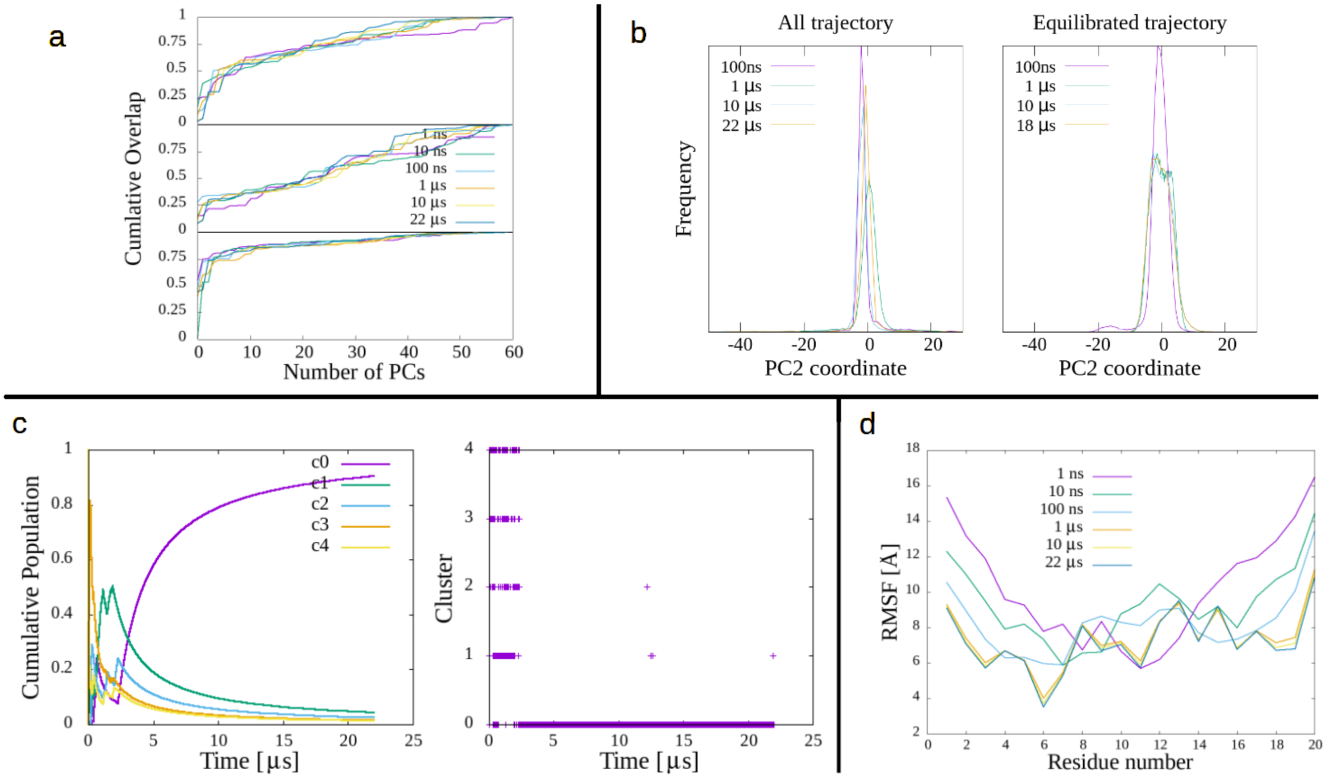

Supplementary Figure 2. Metrics of convergence in Trp-cage. A) Cumulative overlap of first 3 three ANM modes, in terms of MD PCs, for several simulation times. B) Frequency distribution of selected PCs for several simulation times. C) Cluster cumulative population (left) and time-distribution (right). D) Residue root-mean square fluctuations for several simulation times.

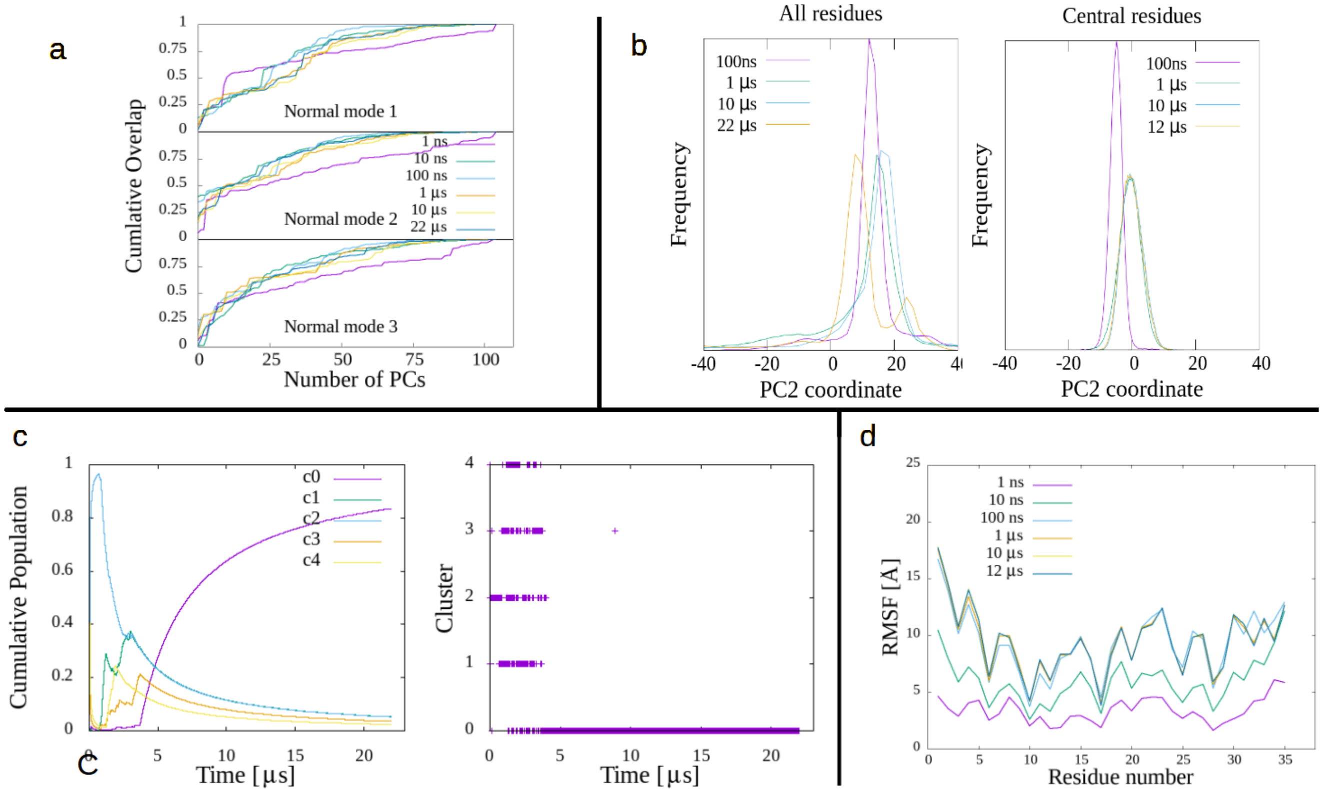

Supplementary Figure 3. Metrics of convergence in VHP. A) Cumulative overlap of first 3 three ANM modes, in terms of MD PCs, for several simulation times. B) Frequency distribution of selected PCs for several simulation times. C) Cluster cumulative population (left) and time-distribution (right). D) Residue root-mean square fluctuations for several simulation times.

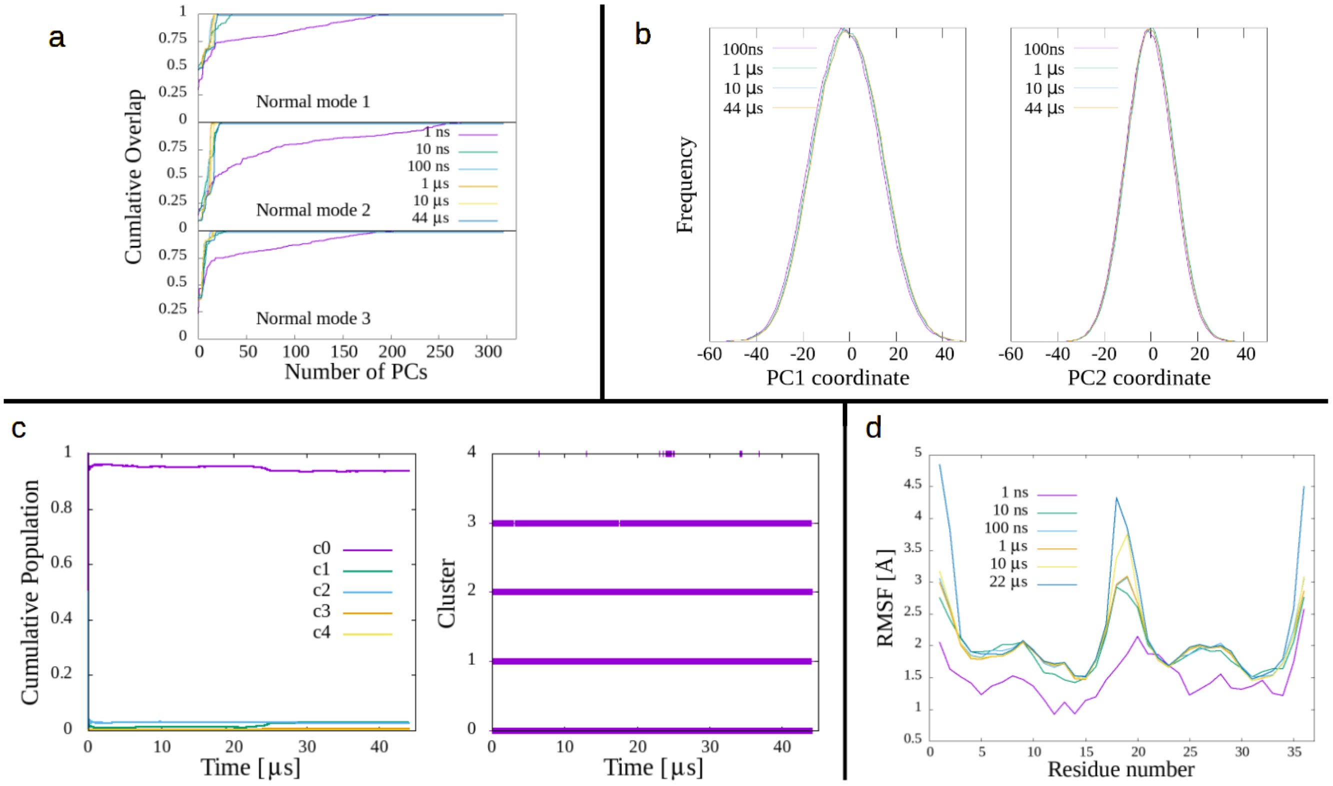

Supplementary Figure 4. Metrics of convergence in GAAC. A) Cumulative overlap of first 3 three ANM modes, in terms of MD PCs, for several simulation times. B) Frequency distribution of selected PCs for several simulation times. C) Cluster cumulative population (left) and time-distribution (right). D) Residue root-mean square fluctuations for several simulation times.

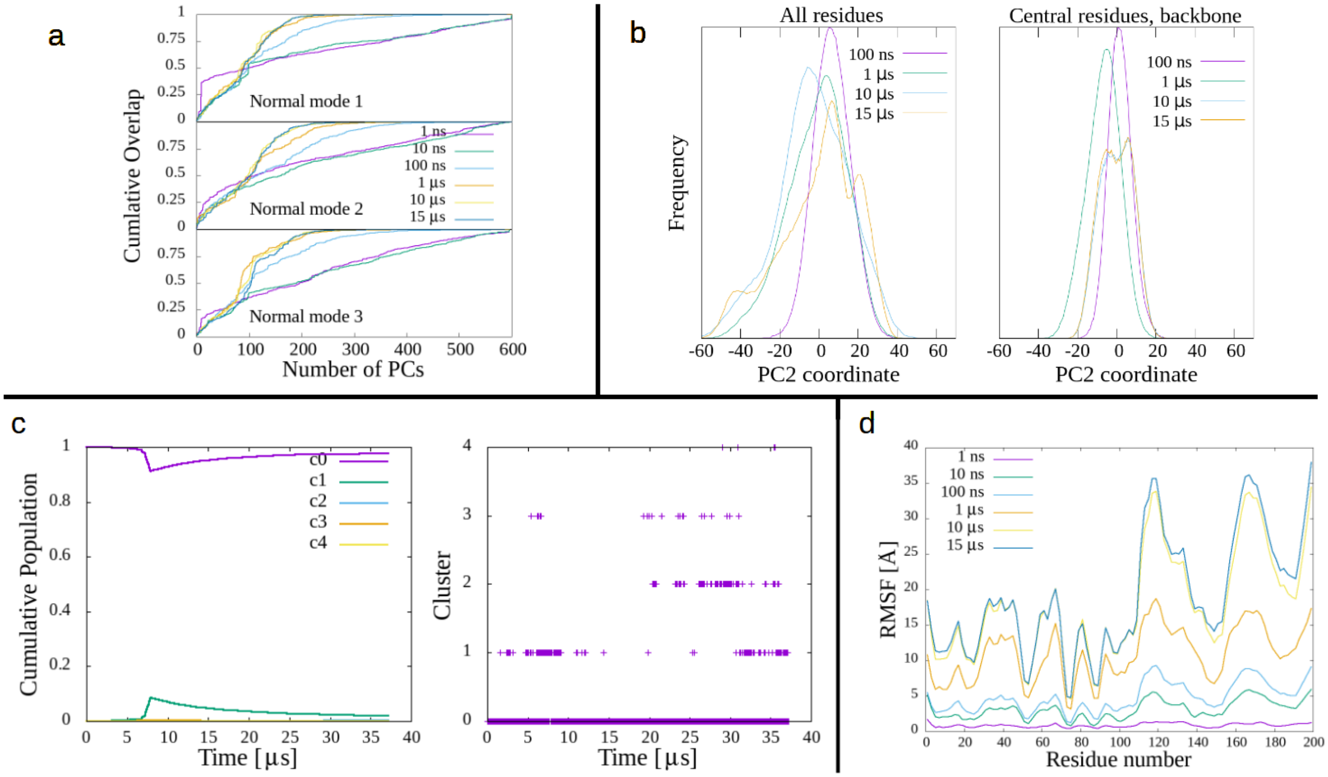

Supplementary Figure 5. Metrics of convergence in barnase. A) Cumulative overlap of first 3 three ANM modes, in terms of MD PCs, for several simulation times. B) Frequency distribution of selected PCs for several simulation times. C) Cluster cumulative population (left) and time-distribution (right). D) Residue root-mean square fluctuations for several simulation times.

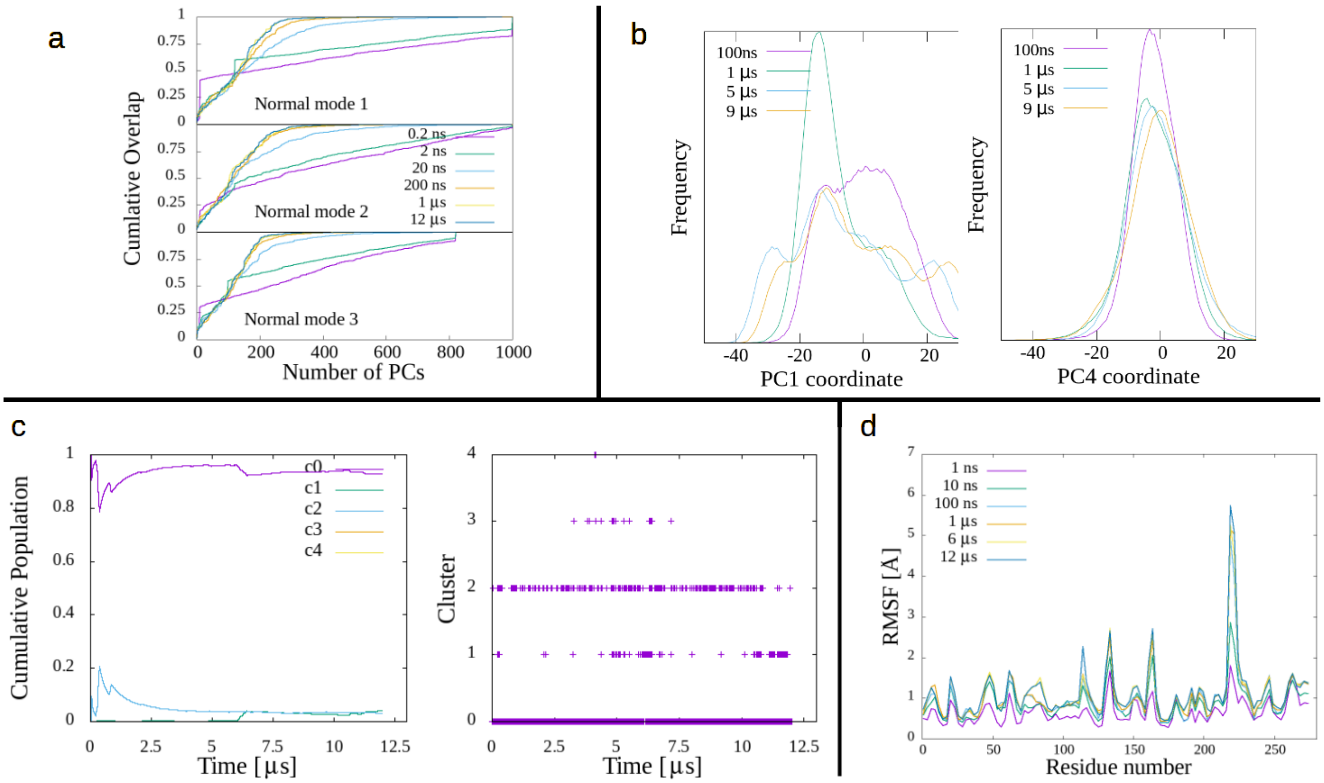

Supplementary Figure 6. Metrics of convergence in elastase. A) Cumulative overlap of first 3 three ANM modes, in terms of MD PCs, for several simulation times. B) Frequency distribution of selected PCs for several simulation times. C) Cluster cumulative population (left) and time-distribution (right). D) Residue root-mean square fluctuations for several simulation times.
