# Supplementary material for: Convergence and equilibrium in molecular dynamics simulations
Source: Commun Chem. 2024 Feb 7;7:26. doi: 10.1038/s42004-024-01114-5 (PMC10850365; doi:10.1038/s42004-024-01114-5)
Supplement: Supplementary file 3 — Description of Additional Supplementary Files [file 42004_2024_1114_MOESM3_ESM.pdf]

Description of Additional Supplementary Files

File name: Supplementary Data 1

Description: Dialanine initial structure

File name: Supplementary Data 2

Description: Dialanine final structure

File name: Supplementary Data 3

Description: Trp-cage initial structure

File name: Supplementary Data 4

Description: Trp-cage final structure

File name: Supplementary Data 5

Description: VHP initial structure

File name: Supplementary Data 6

Description: VHP initial structure

File name: Supplementary Data 7

Description: GAAC initial structure

File name: Supplementary Data 8

Description: GAAC final structure

File name: Supplementary Data 9

Description: Barnase initial structure

File name: Supplementary Data 10

Description: Barnase final structure

File name: Supplementary Data 11

Description: Elastase initial structure

File name: Supplementary Data 12

Description: Elastase final structure

File name: Supplementary Data 13

Description: PGK initial structure

File name: Supplementary Data 14

Description: PGK final structure

File name: Supplementary Data 15

Description: $3CL^{pro}$ initial structure

File name: Supplementary Data 16

Description: $3CL^{pro}$ final structure

File name: Supplementary Movie 1

Description: Dialanine trajectory

File name: Supplementary Movie 2

Description: PGK trajectory

File name: Supplementary Movie 3

Description: $3CL^{pro}$ trajectory

File name: Supplementary Movie 4

Description: Trp-cage trajectory

File name: Supplementary Movie 5

Description: VHP trajectory

File name: Supplementary Movie 6

Description: GAAC trajectory

File name: Supplementary Movie 7

Description: Barnase trajectory

File name: Supplementary Movie 8

Description: Elastase trajectory
